# Analysis of Effectiveness of a Supplement Combining *Harpagophytum procumbens*, *Zingiber officinale* and *Bixa orellana* in Healthy Recreational Runners with Self-Reported Knee Pain: A Pilot, Randomized, Triple-Blind, Placebo-Controlled Trial

**DOI:** 10.3390/ijerph18115538

**Published:** 2021-05-22

**Authors:** Marcela González-Gross, Carlos Quesada-González, Javier Rueda, Manuel Sillero-Quintana, Nicolas Issaly, Angel Enrique Díaz, Eva Gesteiro, David Escobar-Toledo, Rafael Torres-Peralta, Marc Roller, Amelia Guadalupe-Grau

**Affiliations:** 1 ImFINE Research Group, Department of Health and Human Performance, Universidad Politécnica de Madrid, 28040 Madrid, Spain; carlos.quesada@upm.es (C.Q.-G.); enrique.diaz@aepsad.gob.es (A.E.D.); eva.gesteiro@upm.es (E.G.); david.escoledo@gmail.com (D.E.-T.); rafaelsanchezdetorres@gmail.com (R.T.-P.); amelia.guadalupe@upm.es (A.G.-G.); 2 CIBER Physiopathology of Obesity and Nutrition (CIBEROBN), Instituto de Salud Carlos III, 28029 Madrid, Spain; 3 Department of Applied Mathematics to Information and Communication Technologies, Universidad Politécnica de Madrid, 28040 Madrid, Spain; 4 Biomechanical Laboratory, Faculty of Physical Activity and Sport Sciences, Universidad Politécnica de Madrid, 28040 Madrid, Spain; javier.rueda7792@gmail.com; 5 Department of Sport, Universidad Politécnica de Madrid, 28040 Madrid, Spain; manuel.sillero@upm.es; 6 Natural Origins, 69380 Lozanne, France; nicolas.issaly@telefonica.net (N.I.); mroller@mynaturalorigins.com (M.R.); 7 Clinical Laboratory Unit, Department of Sport and Health, Spanish Agency for Health Protection in Sport (AEPSAD), 28040 Madrid, Spain

**Keywords:** knee joint inflammation, recreational runners, biomechanical phenomena, thermography, *Harpagophytum procumbens*, *Zingiber officinale*, *Bixa orellana*

## Abstract

Recreational running (RR) is becoming a popular way to increase physical activity for improving health, together with a higher incidence of knee injuries. The aim was to analyze the effect of a four-week supplementation with a mixture of *Harpagophytum procumbens*, *Zingiber officinale and Bixa orellana* on males, middle-aged, RR with an undiagnosed knee discomfort. A randomized triple-blind placebo-control trial was conducted among male RR aged 40–60 years suffering from self-declared knee discomfort after training. Participants were assigned to supplementation (2 g/day in 6 doses; *n*  =  13; intervention group (IG)) or matched placebo (*n*  =  15; control group (CG)) for 4 weeks. At pre- and post-intervention, assessment of routine blood biomarkers, body composition, running biomechanics and body temperature was performed using standardized procedures. Machine learning (ML) techniques were used to classify whether subjects belonged to IG or CG. ML model was able to correctly classify individuals as IG or CG with a median accuracy of 0.857. Leg fat mass decreased significantly (*p* = 0.037) and a deeper reduction in knee thermograms was observed in IG (*p* < 0.05). Safety evaluation revealed no significant differences in the rest of parameters studied. Subjects belonging to IG or CG are clearly differentiated, pointing into an effect of the supplement of ameliorating inflammation.

## 1. Introduction

During the last few decades, physical activity (PA) in all domains (work, housework, transport, leisure time) [1] has been decreasing [2], with a proven negative impact on health [3]. From all domains, leisure time is one of the most feasible domains to be increased [4]. This assumption is the baseline for current public health strategies, encouraging people to reach the recommendations of the World Health Organization, published in their Global Strategy on Diet, Physical Activity and Health, and in the just updated guidelines [5]. One of the most popular options to fulfill this goal is recreational running (RR). RR is currently a regular PA for millions of people around the world. Recreational runners are estimated to be around 49.9 million in Europe alone [6]. It has been suggested that this prevalence may be due to RR characteristics as it allows for social interaction, it may be fun while still allowing the possibility of competition, and it is comparatively cheap as well as relatively simple [6,7,8,9]. 

Even assuming the well-described benefits [10], exercise by itself can increase the risk of injuries. Interestingly, recreational running is (RR) even more prone to the appearance of injuries than some other available exercise options [11]. An increased running-related injury incidence of 7.7 per 1000 h of RR (95% CI: 6.9–8.7) has been reported [12]. The lower limb is the part of the body more prone to suffer injuries in these subjects, the knee being the most frequent site [13]. Injuries are mostly associated with patellofemoral dysfunction syndrome (PFDS) or patellofemoral pain syndrome (PFPS) and are even called “runner’s knee” in medical literature, usually associated with *Chondromalacia patellae*. It is one of the most prevalent overuse disorders involving this region and the most common chronic injury and specific pathology recorded in RR [11,14]. 

Although there are many different etiologies and pathologies, a common symptom to most forms of joint disease is the presence of joint inflammation and discomfort associated with overuse. Overuse injury has been defined as a micro-traumatic damage to a body tissue that has been under repetitive stress without sufficient time to go through the natural repairing process [15]. Altogether, overuse disorders in RR during the training season may affect up to 48.3% of the runners of a popular race. Curiously, only 14.2% of them reported having asked for medical aid during or after the race [16]. Furthermore, several runners will even maintain their training schedule despite reporting joint discomfort after or, less usually, during the training sessions. From a total of 1145 novice and recreational runners analysed in a study in the UK, 570 were injured, from whom 86%, suffered pain directly affecting their performance and reducing their running volume, but continued running [17]. Although there is a variety of treatments available for these types of injuries, mainly anti-inflammatory and/or analgesic drugs, many solutions are not proving to be long-lasting for some subjects, despite the intense research on the topic [18,19]. 

At earlier stages of pain, runners may decide to take dietetic supplements as complementary treatments to help them endure the roughness of the intense PA without decreasing their performance [20,21]. Herbal products on the market being used as alternatives to NSAIDs are mostly extracts from seeds, roots, leaves, barks, or berries. Botanicals, either whole powders or extracts from part of plants are well accepted by most recreational runners. *Harpagophytum procumbens* and *Harpagophytum zeyheri,* both known as *devil´s claw*, have been extensively used against pain and usually have a good response among this population. *Harpagophytum* products have shown efficacy in osteoarthritis treatment and low back [22,23], up to 8 weeks of supplementation [24]. In fact, the potential role of devil’s claw in managing inflammation and oxidative stress-related diseases has been reviewed in preclinical and clinical pathological conditions in which inflammation plays a key causative role [25]. Since the synergistic effect of devil’s claw with other herbal preparations has been suggested, there is an intense search for a synergistic action with other herbal products in order to provoke an ergogenic effect [26]. 

Due to the long-term effects of joint inflammation on running biomechanics, it could be assumed that a relative decrease of knee joint inflammation would cause a beneficial effect on RR, which could be ergogenic for some types of efforts, especially long duration races. The analgesic and ergogenic aid of ginger in sport (*Zingiber officinale*) has been reviewed by Wilson in 2015 [27]. Ginger’s bioactive compounds have been stated as anti-inflammatory products through the inhibition of macrophages and neutrophils activation [28,29,30]. In sport, ginger has been used as an ergogenic aid and also for its analgesic properties, as an alternative to the use of nonsteroidal anti-inflammatory drugs [27]. Furthermore, a five-day supplementation of ginger root was shown to have moderate beneficial effects on running-induced soreness of recreational runners [31]. In fact, ginger is being used in osteoarthritis and to mitigate post-exercise soreness [32,33]. An herbal mixture of ginger root and annatto (*Bixa orellana*) seed supplementation for 4 weeks has been shown to decrease the pain associated with delayed onset of muscle soreness [34]. Bixin and norbixin, the main carotenoids present in annatto seed, were shown to decrease oxidative stress and levels of inflammatory cytokines in healthy subjects [35].

Therefore, considering the anti-inflammatory, ergogenic and analgesic properties of the three botanicals described above, and supposing their potential synergistic effects, the aim of our study was to analyze a four-week supplementation intervention in the alleviation of symptoms, running biomechanics, blood parameters, body composition and body temperature in male, middle-aged, recreational runners with an undiagnosed knee discomfort.

## 2. Materials and Methods

### 2.1. Subjects

Participants were recruited through the “snowball” method, starting by contacting local athletics clubs in the Madrid area (Spain) to introduce the study. Social media were used to spread the information to the target population. An e-mail to a specific account created for the purpose allowed the selection of participants. Inclusion criteria were: (1) male; (2) aged 40–60 years, with undiagnosed significant knee pain after training; (3) scoring ≥2 on a Visual Analogue Scale (VAS); (4) training weekly (>150 min of exercise per week divided into two or more days). Physical Activity Readiness Questionnaire (PAR-Q), a health questionnaire, was used as a first step for inclusion, aiming to accept only subjects capable of performing a maximal test to exhaustion without risks. Exclusion criteria were smoking; suffering from any chronic disease or impairment to perform exhausting exercise; diagnosed knee injury or having undertaken an operation; use of dietary or herbal supplements at the onset of the study; use of any anti-inflammatory or analgesic drugs at the onset of the study or the previous month or the use of antibiotics three months before the intervention. As this trial was conducted as a pilot approach to a potentially larger trial, it was agreed upon 30 subjects as it is a generally accepted number for pilot studies. The power of the contrasts was studied *a posteriori* and the sample size necessary for inference was then calculated with G*Power software (version 3.1; Heinrich-Heine-Universität Düsseldorf, Düsseldorf, Germany) with reference to the variability of the dependent variable (mean temperature of the knee) for a power of 80%. 

Thirty-three subjects fulfilled the inclusion criteria. All participants signed an informed consent to take part in the study and were randomly assigned to the matched placebo (control group, CG) [36] or the intervention group (IG). Due to several reasons explained in the results section, some subjects had to be excluded and the final sample was composed of 13 subjects in the IG and 15 in the CG. None of the research personnel who enrolled, treated or assessed the subjects was aware of the assignments until the triple-blind study was complete. 

The study was performed according to the last update of the Helsinki Declaration (2013) and approved by the Ethical Committee of the Universidad Politécnica de Madrid (approval number 20180730-1). Written informed consent was obtained from all the included participants. National and European legislation related to data protection was followed rigorously. The study was registered at clinicaltrials.gov (NCT04150211).

### 2.2. Study Protocol

**Figure 1:**
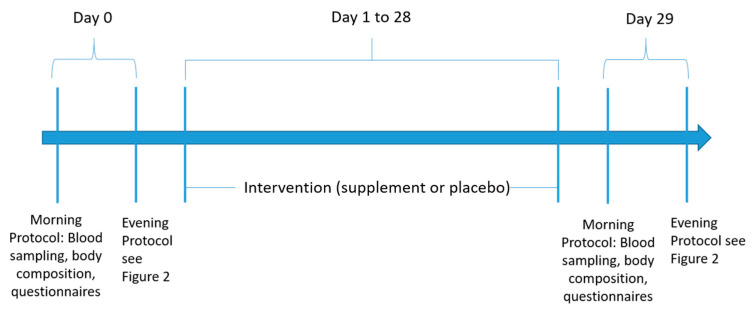
Schematic view of the study.

Volunteers for the study had to be physically present at the research facilities on four occasions, on two days, as there were two visits each day, one in the morning and one in the evening. All the tests were performed following the same order for all participants and at the same relative time distance. Both testing days were separated by 30 days. Meanwhile, the subjects were encouraged to continue with the same training and dietary habits as previously stated, adding only the ingestion of supplement or placebo. For that purpose, participants received a blank box, labeled only with their research ID number, on the first visit. It contained the amount to allow the daily ingestion of either 2g of Exten(d) (2 capsules with breakfast, 2 with lunch and 2 with dinner) or the same number and type of capsules filled with 2 g of placebo. The schematic view of the protocol is shown in Figure 1.

#### 2.2.1. Mornings

Participants arrived early morning and in a fasting condition. Body composition and blood extraction were performed. Afterwards, dietary, PA and lifestyle questionnaires were filled in by a trained researcher. Standardized breakfast was provided for participants and advice regarding the previous meal to the evening part of the experiment was provided.

#### 2.2.2. Evenings

**Figure 2:**
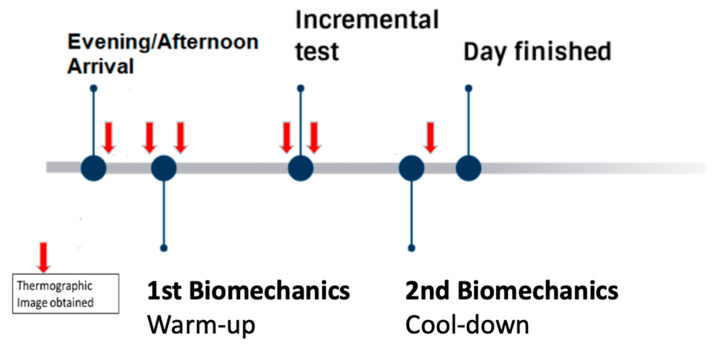
Schematic view of the evening protocol.

After arrival at a fixed time, all participants had infrared thermography images [5] of their lower limbs taken at the Biomechanics Laboratory, previously and just after a test of running technique and biomechanics. Afterwards, they were medically examined before performing an incremental exercise test to exhaustion on a treadmill (IE) inside the Physiology Laboratory. Again, IRT were taken just before and after the IE. As soon as possible after the fatiguing IE, subjects performed another test of running technique at the Biomechanics Laboratory to assess possible changes and another IRT was taken once the test was finished (Figure 2).

### 2.3. Socioeconomic Data and Lifestyle Behavior

At the beginning of the study, a general survey was filled in by each participant in order to record their personal data (part of the validated EXERNET General Questionnaire 3.0) [37] including age, sex, socioeconomic factors, lifelong sports habits, sleep patterns, health condition and lifestyle. 

### 2.4. Physical Activity

At the beginning and the end of the study, subjects filled in two International Physical Activity Questionnaires (IPAQ) [38], a validated and self-administered questionnaire for people aged 15–69 that recorded PA habits at work, during transportation, at home and doing sport and physical exercise, sitting and leisure time. Results are given in Metabolic Equivalents (METs). Participants were asked not to change their usual training schedule during the intervention. It was complemented by a brief record of exercise performed during the month of intervention detailing the type of exercise, hours per week, mean intensity, and review of competitions, if any, in order to make sure that PA levels were maintained throughout the study.

### 2.5. Dietary Intake

Participants were interviewed by a trained professional nutritionist using the 24-h dietary recall method with the support of a booklet including photos of different portion sizes of usual food and drinks in Spain [39], before and after the intervention. The nutritional composition of the diet was analyzed using Dial Software© (Alce Ingeniería, Madrid, Spain).

### 2.6. Anthropometry

Height was collected by a well-trained ISAK-certified researcher using a portable stadiometer (SECA, Hamburg, Germany). Weight was measured by means of electric bioimpedance (BC-418MA, Tanita Corp., Tokyo, Japan). For all participants, 0.6 kg were subtracted for the total weight in order to correct the clothes weight. BMI was calculated using the formula BMI = weight (kg)/height (m)^2^. 

Body composition was determined early in the morning in fasting condition by dual-energy x-ray absorptiometry (DEXA) (Lunar Prodigy; GE Healthcare, Madison, WI, USA). From the DEXA scans, the trunk and limb specific composition were obtained.

### 2.7. Clinical Chemistry

Fasting blood samples were collected by qualified nurses in the Clinical Laboratory unit at the Department of Sport and Health. Spanish Agency for Health Protection in Sport (AEPSAD). Complete blood counts were determined from Vacutainer^TM^ EDTA tubes (Becton Dickinson, Franklin Lakes, NY, USA) by standardized clinical laboratory procedures using an automated hematology analyzer (ADVIA 120; Siemens Healthcare Diagnostics Inc., Deerfield, IL, USA) within 4h after extraction. For serum separation, blood samples were collected in Vacutainer^TM^ gel tubes (No additive (Z) [40,41,42] (Becton Dickinson, Franklin Lakes, NY, USA). Samples were stored at room temperature for at least 15 min to allow clot formation and then immediately centrifuged for 15 min at 4000 rpm and 10 °C. Serum samples were aliquoted and analyzed to assess basic biochemistry in an AU400 analyzer (Beckman Coulter Inc., Brea, CA, USA) and stored at −80 °C for later analysis.

### 2.8. Thermography

Thermograms were recorded with a FLIR T530 camera (FLIR Systems, Täby, Sweden) with 320 × 240 pixels, NETD < 40 mK, and a temperature range of −20 to 100 °C, following the statements of the Thermographic Imaging in Sports and Exercise Medicine (TISEM) consensus document, setting the emissivity of the camera on 0.98, corresponding to the skin temperature. The camera was turned on at least 10 min before the beginning of the data collection to allow self-calibration and sensor stabilization. During each visit, six rounds of thermograms were taken: basal round (IRT0), after a 20-min acclimation to room temperature (IRT1), after first biomechanical running test (IRT2), before incremental test to exhaustion (IRT3), immediately after incremental exercise test to exhaustion (IRT4) and after cooling down (IRT5). 

**Figure 3:**
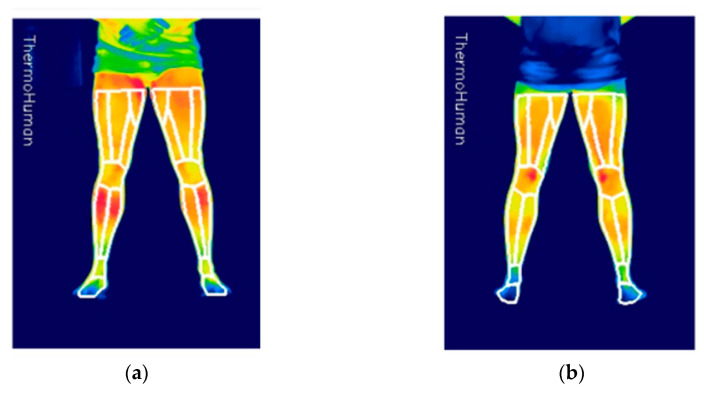
Thermographic imaging. (**a**) Front and (**b**) back.

Each round consisted of four thermograms taken from a fixed distance by the same trained researcher (anterior and posterior lower limbs) (Figure 3).

Subjects observed the standard preparatory protocol for thermal imaging measurement [41]. The standardization of a number of factors, included joint positioning, environmental temperature, placing of the limb in relation to the IRT device and the determination of the region of interest (ROIs) for acquisition of cutaneous temperature data, were all established and controlled by an expert, according to the methodology proposed by Fernández-Cuevas et al. (2015) [43]. Average room temperature in the laboratory was 24.6 ± 0.5 °C. The subjects acclimated to the room temperature at least for 20 min while clinical exploration took place before the first image was taken.

Thermograms were analyzed with validated Thermohuman Software^©^ (Thermohuman, Madrid, Spain) for automatically extracting the minimum, maximum and averaged skin temperatures of 18 preselected ROIs in each image, from which only the 6 ROIs corresponding to the legs and knees were used [44]. Ankles and thighs were discarded in order to avoid problems in the thermal images due to the shorts used by some participants, and due to sneakers and socks. The biomechanical image markers were ignored by the software as they were not inside any ROI. This fact was specifically confirmed taking thermograms before the positioning of the markers and after it. 

Front (FK) and back (BK) of the knee joint, front leg (FL) and back leg (BL) (i.e., patella, popliteal fossa, superficial ankle extensor muscles and superficial ankle flexor muscles, respectively) were analyzed. The software automatically provided the average skin temperature (Tsk) and the number of pixels (n) for each ROI. In case of the areas composed for more than one ROI (i.e., FL and BL), the two corresponding ROIs provided by Thermohuman and were averaged (TskTOT) in both sides in order to be used in the statistical analysis as a single ROI using the Equation (1):

TskTOT = ((Tsk1 × n1) + (Tsk2 × n2))/(n1 + n2)(1)

### 2.9. Biomechanics

A system composed of 6 infra-red cameras (VICON, Yarnton, Oxfordshire) recording at 120 Hz and two dynamometric platforms (Kistler, Winterthur, Switzerland) operating at 1000 Hz were used to measure reaction forces and to determine the events of foot contact and foot off while subjects ran on a synthetic floor indoor lane of 15 m. Researchers helped to set the pace of running at 3 m/s. A total of 38 reflective markers (32 for the dynamic captures) at shoulders, arms, pelvis, thighs, lower legs and feet were placed on each subject while their skin adjusted skin to room temperature after arrival and basal IRT.

To simplify the analysis, the left leg was randomly selected for this study, rather than calculating the mean of both legs. This approach was preferred in order to use real data of the participants rather than the use of an estimation.

### 2.10. Ergospirometry

After the first biomechanics test, the subjects cooled down for over 30 min; meanwhile, a specialized and experimented sports physician examined each participant. Anamnesis and basal 12-lead electrocardiogram (ECG) to confirm health status were performed immediately before the IE in the Physiology Laboratory. Supervised and encouraged running IE on a computerized treadmill (CH/P/Cosmos 3PW 4.0; H/P/Cosmos Sport & Medical, Nussdorf-Trauntein, Germany) was performed starting with a three-minute warm-up at 10 km/h (Figure 1). The speed increased 0.2 km/h every 6 s until exhaustion. Heart rate (HR) was continuously monitored using a 12-lead ECG. Expired gases were measured breath by breath in a Jaeger Oxycon Pro gas analyzer (Erich Jaeger, Viasys Healthcare, Höchberg, Germany). Subjects either gestured in order to request for the test to be concluded or simply stepped out of the treadmill if they could not sustain the effort any longer. Then, the treadmill speed was reduced to stage 1 and maintained for two minutes in order to “cool down” and completely stopped afterwards. In all cases, either the maximal HR (30 s average, >220–age) or the respiratory exchange ratio (RER) of less than 1.1 was reached. This test was conducted the first day as baseline and repeated after 30 days of supplementation.

### 2.11. Composition of the Supplement

The blend used was a commercialized product called Exten(d), blend 1/0.75/0.375 (*w*/*w*/*w*), supplied by Natural Origins (Lozanne, France). It is a blend of *Harpagophytum procumbens* with *Harpagophytum zeyheri* Decne root powder, (product code #3OPH063004) with *Zingiber officinale* Roscoe rhizome powder (product code #3OPH056912) and *Bixa orellana* L. seed powder (#CPH050872). The analysis of the powders used through high performance liquid chromatography (HPLC) showed a total iridoid content of 1.65% (*w*/*w*) as the sum of harpagoside (1.22%), harpagide, 8-p-coumaroyl harpagide (8PHPG) and two others minor iridoids. The occurrence of gingerols and shogaols contents in ginger was set to 2.4% (*w*/*w*), expressed as capsaicin. The total bixin and norbixin was 1.32% (*w*/*w*). Placebo capsules were indistinguishably packaged and labeled. The content was the same weight of maltodextrin (#1OPC057321 Natural Origins, France). Capsules containing Exten(d) or placebo were supplied by NovaPharm Laboratoires (Vendargues, France). All the capsules had to be swallowed whole to avoid flavor or texture differences being perceived.

### 2.12. Statistical Analysis

As described above, data used for the analysis came from various origins; ergospirometry, clinical chemistry, DEXAs, thermography and biomechanics, each generating a different dataset. In all cases, the datasets contain longitudinal data and are divided in two groups: IG and CG. More precisely, each dataset contains 56 samples, composed of 13 individuals included in the IG and 15 in the CG, for which all measures were taken twice, pre- and post-intervention. Missing values accounted for 5% of the total data, but they were distributed unevenly; the great majority of variables showed <1% missing values, while some variables had >70%. Variables with a high incidence of missing values were discarded from the study while the rest was preprocessed using the median of each variable for non-analyzed imputation.

Statistical analysis was divided in two main parts. A first descriptive study was conducted to find significant pre- and post-intervention intragroup differences for each of the variables as well as to check for intergroup differences. As for the intragroup tests, significant pre-post intervention differences were assessed for all variables by means of paired t-tests. Normality was checked using Shapiro–Wilk tests and homoscedasticity by means of Levene’s test. When the latter was violated, Welch correction was applied to the paired *t*-test. Kruskal–Wallis tests were performed in non-normality outcomes. The intergroup tests were conducted in a similar way, using the non-paired version of each of the tests, as individuals are different in the IG and CG. 

More intricate relations might be overlooked by these kinds of methods, specifically when (possibly nonlinear) combinations of the variables are related to membership to each group. This motivates the second part of the statistical analysis, conducting classification methods of machine learning [45] with the purpose of checking whether a subject can be identified to be inside the control or the intervention group by means of the rest of the variables. The idea behind using classification methods is the following: if it is possible to accurately predict whether a subject is in the CG/IG by just looking at the measurements, then the intervention is producing different outcomes for subjects in a group compared to those in the other group.

The main advantage of this approach is that classical hypothesis testing looks for linear relations such as differences in means (t-test) or medians (Kruskal–Wallis). In fact, if no distributional assumption is to be considered then only dominance of a distribution over another is inferred. Further, the effects of the groups on several variables are considered individually. Significant nonlinear relations between the intervention/control variable and one [or more] variables might be occurring and with the help of a classifier we may be able to find this pattern.

A difficulty arises regarding these nonlinear models when trying to measure how well we are describing the ground truth. A very complex model could potentially detect very specific properties of a particular dataset without having any generalization power as it has focused so much on that task, but it might not be accurate on new observations. This situation is called overfitting and is solved using cross-validation.

A second problem is that machine learning algorithms frequently act as a black box [46], making it cumbersome to detect which variables had a bigger impact on the results. Few exceptions feature model-specific metrics for measuring this importance. For classifiers without a built-in method for measuring the importance, a filter approach is used, conducting receiver operating characteristic (ROC) curve analysis for each of the variables [47].

All five datasets were combined into a single data frame of 56 observations and 890, falling into the high dimensional low sample size paradigm [48]. In this situation, where the number of variables is clearly larger than the number of observations, the risk of overfitting becomes even more critical. Two of the main ways of overcoming this problem are to use filter methods that reduce the dimensionality of the dataset before applying any algorithm and using ensemble methods. Both strategies can in fact be combined. The main idea of an ensemble methodology is to combine a set of models, each of which solves the same original task. In particular, bagging methods, random forests and boosting have proven particularly useful [49]. In this study, we used the InfoGain filter [50] to create a reduced dataset followed by the Deepboosting algorithm, an ensemble version of the widely known tree classifiers [51].

All the statistical analysis was conducted using SPSS 25.0 (released in 2017; IBM Corp., Armonk, NY, USA) and R version 4.0.2 under the frontend RStudio 1.3.1056. The first part was calculated using only functions from the default libraries from R. The InfoGain filter was computed by means of the FSelector package [52] and the Deepboosting model was trained using the caret package [47], validating the results by means of 200 group Monte Carlo cross validations with non-overlapping patients so that the accuracy is not over optimistic, using 75% of the observations for the train. Additionally, the library data table was used to perform much of the preprocessing [53] and doParallel was used for parallelization.

## 3. Results

From the thirty-three subjects recruited for this experiment, three did not come to the second visit (*n* = 2) due to injury and (*n* = 1) due to medical reasons not related with the trial. From the 30 subjects who completed the study, two had to be withdrawn from the IG (one because of gastrointestinal problems not related with the trial and the other because of an injury that occurred during the final IE). Overall, treatments were well tolerated. An adverse event appeared in the form of persistent gastrointestinal discomfort in one of the subjects receiving the supplement, but none on the placebo, as could be expected. However, the discomfort was not severe, since it allowed the subject to complete the month of supplementation and to finish the trial.

All subjects completing the protocol presented normal ECG and arterial blood pressure at rest (data are not shown). Descriptive characteristics of subjects are shown on Table 1. Due to random triple-blind assignment to IG or CG, there was a small but significant difference in age between groups (*p* < 0.05), hence the use of groups of age and body mass index (BMI) for the final stage of statistical analysis. Routine blood hematology and biochemistry and treadmill tests were used as safety biomarkers to assess possible negative effects according to the National Cancer Institute Common Terminology Criteria for Adverse Events (CTCAE) v. 4.0. Administration of 2 g of Exten(d) for 30 days did not result in any significant differences in clinical chemistry parameters measured as compared to the changes caused by the maltodextrin control. Only a significant (*p* < 0.05) intragroup increase in aspartate transaminase (GOT) was observed in the CG; however, this outcome has no physiological significance as values are within the normal range. No performance difference between groups was observed during the IE (Table 1). For the rest of characteristics, no differences were observed (data not shown). 

### 3.1. Body Composition

No difference between and within groups was observed for body composition during the study (Table 1). There was only a significant decrease in fat mass of the legs (%) in the IG (from 4.84 ± 1.39 to 4.62 ± 1.71 kg, *p* = 0.036). No difference was found between groups as well as no changes intragroup detected for the rest of variables.

### 3.2. Thermography

**Figure 4:**
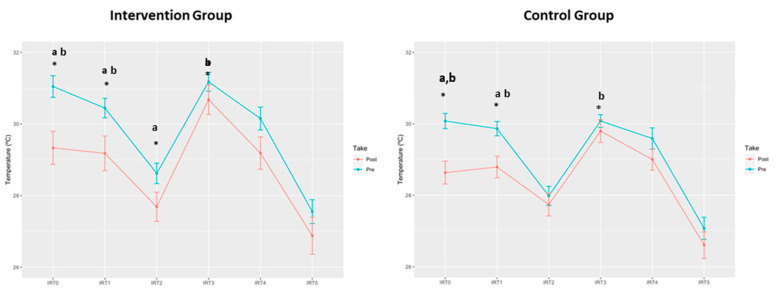
Skin temperature (°C) before (Pre) and after (Post) administration of 2 g of Exten(d) for 30 days to volunteers compared to placebo. IRT0 = basal round; IRT1 = after acclimation to room temperature; IRT2 = after first biomechanical running test; IRT3 = shortly before incremental test to exhaustion; IRT4 = immediately post-incremental exercise test to exhaustion; IRT5 = after cooling down. * *p* < 0.05; a = intragroup; b = intergroup (Pre).

Significant differences on the knee Tsk were obtained in the moments T0, T1 and T2 in the IG and for T0 and T1 in the CG, being lower after the intervention in both groups (Table 2, Figure 4). Probably due to the triple blind randomization, mean knee temperatures before the intervention were higher in the IG than in the CG, being the differences significant between groups at T0, T1 and T3. After the intervention, these significant differences disappeared. This means that there was a significant reduction of knee Tsk in the IG which could not be observed in the CG. No significant results were found for thermographic results on the anterior and posterior leg.

### 3.3. Biomechanics

IG started this research with a significantly lower gait frequency than CG (*p* = 0.028), which increased nearly significantly (*p* < 0.058) after the intervention (Table 3). The intragroup analysis of IG also showed a significant increase in hip adduction (*p* = 0.036) and a nearly significant increase in max. hip extension during foot contact (*p* = 0.086) between day 0 and day 30. In the intergroup analysis after the intervention (T1), IG showed a longer foot contact time (*p* = 0.098) and a lower knee flexion at foot contact than the CG (*p* = 0.068). However, these differences were non-significant and, even if no change was observed in max. Knee flexion in both the contact and the swing phases, valgus and rotation variables, different patterns of knee flexion during foot contact and during swing phase were observed between groups. No significant differences were found in CG between the two test days.

### 3.4. Machine Learning Analysis

The combined approach of using a filter method together with the Deepboost ensemble algorithm showed powerful results; the model was able to capture significant differences between the IG and CG when considering all variables at once. The reduced dataset consisted of 150 variables selected by the filter method. The choice of restricting the dataset specifically to 150 variables was made by trial and error. Several other choices were tried and all lead to worse results. The Deepboost classifier was applied using 1 as tree depth, a logistic loss, and parameters λ = 0.25, β = 0.005, and they can be understood as follows. The first parameter is the depth of the trees that are being ensembled, the logistic loss is used since we are dealing with a dichotomous prediction and λ, β, account for regularization that the algorithm uses; that is, we tell the algorithm not to learn too many details of the dataset so that it has a better generalization capacity. Again, all parameters were selected after trying several choices and keeping the best one, according to cross-validation. With these choices, the model could predict whether a new observation belonged to the IG or the CG with a median accuracy of 0.857, while the more conservative kappa statistic was 0.696. All 150 variables selected by the filter belong to the DEXAs, thermography or biomechanics, indicating that those techniques are the ones where the supplement is perceived. More importantly, the study of variable importance showed that the most decisive variables when classifying were variables that come from the thermography, and more specifically the variables related to the knee (and not so strongly, popliteus). This is consistent with the results presented in Section 3.2. 

## 4. Discussion

A randomized triple-blind placebo-control pilot trial was conducted to analyse the effect of a four-week supplementation with a mixture of *Harpagophytum procumbens, Zingiber officinale and Bixa orellana* among middle-aged male with an undiagnosed knee discomfort. The results of our study suggest that the combination of *Harpagophytum procumbens* with *Harpagophytum zeyheri* Decne root powder, *Zingiber officinale* Roscoe rhizome powder and *Bixa orellana* L. could be a potential supplementation for a recreational runner with undiagnosed knee pain after training. The machine learning (ML) approach allowed to clearly distinguish whether the subject belonged to the CG or the IG by taking into account data from DEXA, thermography and biomechanics. This points into a clear effect of the supplement in the IG with respect to the CG. This is supported when data are analysed separately with classical statistics, as we observed significant differences for body composition and thermography in the IG.

Knee joints are particularly stressed while running and several studies have attempted to find non-pharmacological compounds to relieve mild pain in RR. Knee temperature has also been linked to radiographic severity of knee osteoarthritis and it has been used for the objective assessment of the effects of anti-inflammatory arthritis therapies in humans [54]. This study displayed a significant reduction in knee Tsk in the IG after supplementation for the moments T0, T1 and T3, which suggests a different behavior of the vascular response of the IG in the first stages of exercise that could be due to the complementary anti-inflammatory properties of the active compounds of the supplement. Consistent with previous studies, these effects have been described in well-trained endurance runners [30]. In this sense, inhibition of macrophage and neutrophils activation was observed after a six-week administration period of *Zingiber officinale*, mitigating post-exercise soreness. Moreover, the analgesic and ergogenic aid in sport of ginger (*Zingiber officinale*) has been reviewed by Wilson [27]. The main pungent compounds in fresh ginger are a series of homologous phenolic ketones known as gingerols and shogaols which include [6]-gingerol (6-GN), while [8]- and [10]-gingerol occur in smaller quantities. The anti-inflammatory activity of 6-GN has been attributed to the inhibition of pro-inflammatory cytokines and antigen presentation by LPS-activated macrophages. In vitro, 6-GN has shown to inhibit LPS-induced NOS and COX-2 in murine RAW 264.7 cells [55,56]. 

Bixin and norbixin, major carotenoids present in annatto (*Bixa orellana* seeds) are well-known for their antioxidant and anti-inflammatory properties, decreasing postprandial levels of inflammatory interleukines and TNF-α [35]. Ginger has shown beneficial effects in case of osteoarthritis [33]. Recently, Haseeb et al. (2017) proposed the use of *Harpagophytum* in the management of pain and inflammation in osteoarthritis [57]. The efficacy of both acute and chronic supplementation of devil’s claw was shown in a rat model of induced arthritis [58]. In vitro, it has been observed that harpagoside could inhibit interleukin (IL)-6 production from primary human osteoarthritis chondrocytes challenged with IL-1β [57]. Devil’s claw effectiveness in blunting lipopolysaccharide-induced production of inflammatory cytokines has also been shown in mouse macrophages [59]. Other research proposed the possibility of synergistic effects of devil´s claw with other herbal preparations to improve physical and mental quality of life in gonarthritis patients. 

To the best of our knowledge, this is the first time that IRT has been used to analyze anti-inflammation properties of a nutritional supplement in this context. IRT has been used for quantifying the effects of different training loads, since it can evaluate local and systemic cutaneous blood flow adaptation as a function of specific type, intensity and duration of exercise [40,41,42]. It has also been applied in detection of overuse injuries [60,61]. Joint injury and inflammation seem to disrupt the normal symmetry of the thermograms. Furthermore, IRT is becoming a useful, cheap and common method for screening local inflammation in the fields of sports medicine and rehabilitation [14,62,63]. The lack of differences in the legs could point out to a higher specificity of the botanicals used in the intervention on the joints; however, this point could not be determined properly due to the discarded Tsk from thigh areas. More research is needed. 

On contrary, this study was not able to determine clearly significant differences in biomechanical parameters. This could be because the protocol used was standard and not specifically designed for detecting worse biomechanical performance due to knee joint pain. An additional explanation could be that well-trained athletes are able to run optimally even with pain, as has been described earlier [17]. In fact, the results of the biomechanical risk-factor analysis associated with running-related injuries are still inconsistent, as has been shown in a recent systematic review, making the analysis of these factors complicated [64]. 

Any adverse cardiovascular event was reported in our study, contrary to another report published previously [65]. Safety of the supplement was confirmed as non-significant differences were observed for blood biomarkers, confirming results of similar studies in which *Harpagophytum* was mixed with other compounds [66]. Regarding biomarkers, we observed an increase of transaminases (GOT and GPT) among CG. On the contrary, an increase in GOT and GPT has been described in rats after 7 days of intake of a crude extract of *Bixa orellana* [67]. 

The combination of the three botanicals as proposed in the current study could be responsible for the (even mild) early results after only 4 weeks of supplementation. Most studies use 9–12 weeks as an intervention period, as after a longer intervention decrease in inflammation should be more evident [29]. Therefore, we introduced an analysis using G-power software for estimation of sample size and sample effect. Results showed that a sample size of 30 subjects per group would have strengthened the significant differences using traditional statistics in knee Tsk. 

This study has several strengths. The subjects were all mature runners with long experience in athletics. The rigorous field protocol, combining different techniques for producing running fatigue and for analyzing running discomfort and inflammation, assures validity of the data. Possible adverse events were controlled with clinical parameters. However, our study presents some limitations. The triple-blind study, which was thought to be a strength, turned out to be also a limitation as age and BMI differed between groups. Training history of the subjects could not be recorded properly, although we assume that randomization has minimized possible bias. Blood pro-inflammatory markers as cytokines could not be measured due to budget limitations. The Deepboost algorithm does not feature inherent metrics for measuring variable importance, so a filter approach is used. Therefore, variable importance should not be understood as a strict ranking but as a qualitative, yet strong description of which variables were deeply involved in making the prediction. Moreover, the sample size is small. More studies are needed for further results on the effects of this kind of supplementation and about the potential synergistic effects of the active compounds combined in the analysed supplement.

## 5. Conclusions

The results of this pilot study support the evidence for the benefits of the nutritional supplement on recreational runners with undiagnosed knee pain after training. The beneficial effects suggest that the different components included in the supplement may ameliorate the inflammatory state and have a protective effect over the exercising knee. The 4 week-administration was well tolerated by the subjects who showed no adverse events. Further studies are needed to assess the possible synergy among the different botanicals used in determining its overall effectiveness.

## Figures and Tables

**Table 1:** Changes in body composition, maximal oxygen consumption and biochemical variables after administration of 2 g of Exten(d) for 30 days compared to placebo.

Variables	Exten(d) (*n* = 13)		*p*	Control (*n* = 15)		*p*	*p*	*p*
	Day 0	Day 30	Intra	Day 0	Day 30	Intra	Inter T0	Inter T1
**Body composition**								
Height (cm)	174.24 ± 1.57	174.24 ± 1.57	----	173.90 ± 1.83	173.90 ± 1.83	----	0.890	0.890
Weight (kg)	76.83 ± 1.73	76.83 ± 1.73	----	76.35 ± 2.46	76.35 ± 2.46	----	0.880	0.880
BMI (kg/m^2^)	25.35 ± 0.64	25.35 ± 0.64	----	25.15 ± 0.40	25.15 ± 0.40	----	0.800	0.800
Fat Mass (%)	15.57 ± 1.58	15.26 ± 1.52	0.321	15.02 ± 1.56	14.99 ± 1.56	0.914	0.798	0.915
Legs Fat Mass (%)	4.84 ± 0.39	4.62 ± 0.33	**0.036**	4.15 ± 0.44	4.19 ± 0.49	0.630	0.249	0.476
Trunk Fat Mass (%)	8.80 ± 1.05	8.74 ± 1.04	0.768	8.87 ± 0.99	8.86 ± 0.99	0.961	0.965	0.938
**Physical performance**								
VO_2_ max (mL/min)	4330 ± 89.56	4323 ± 90.58	0.700	4067 ± 124.7	4075 ± 93.46	0.89	0.09	0.068
**Biochemical variables**								
Cholesterol (mg/dL)	203.1 ± 5.88	219.6 ± 5.69	**0.070**	213.3 ± 12.2	219.6 ± 15.09	0.182	0.475	0.999
HDL (mg/dL)	63.06 ± 2.57	60.77 ± 2.67	0.110	60.90 ± 2.39	61.67 ± 2.15	0.250	0.560	0.803
LDL (mg/dL)	147.64 ± 4.30	154.23 ± 4.76	0.070	161.55 ± 6.74	171.71 ± 9.44	**0.025**	0.107	0.125
VLDL (mg/dL)	16.24 ± 1.70	15.37 ± 1.27	0.539	19.83 ± 4.64	15.33 ± 1.87	0.178	0.477	0.986
Triglycerides (mg/dL)	81.2 ± 8.52	76.85 ± 6.37	0.539	99.16 ± 23.20	76.64 ± 9.32	0.178	0.477	0.986
GPT (IU/L)	22.31 ± 1.87	22.23 ± 1.42	0.940	19.73 ± 1.49	24.53 ± 2.43	0.170	0.292	0.420
GOT (IU/L)	27.46 ± 2.01	26.69 ± 1.49	0.427	25.87 ± 1.14	29.33 ± 1.53	**0.047**	0.497	0.2278
GGT (IU/L)	42.62 ± 10.75	32.62 ± 6.76	0.107	22.73 ± 2.30	22.67 ± 1.91	0.940	0.093	0.478
Urea (mg/dL)	41.77 ± 2.45	41.61 ± 3.50	0.953	42.41 ± 1.75	40.27 ± 2.46	0.475	0.832	0.758
Uric acid (mg/L)	5.45 ± 0.26	5.33 ± 0.27	0.540	5.76 ± 0.23	5.79 ± 0.21	0.868	0.386	0.201
Albumin (g/dL)	4.40 ± 0.06	4.41 ± 0.08	0.908	4.41 ± 0.04	4.42 ± 0.04	0.774	0.933	0.902
Creatinine (mg/dL)	1.03 ± 0.02	1.01 ± 0.01	0.366	1.07 ± 0.02	1.06 ± 0.02	0.136	0.287	0.167
CK (mU/mL)	232.2 ± 28.8	198.3 ± 27.3	0.257	194.7 ± 26.4	277.1 ± 52.24	0.158	0.348	0.196
Hemoglobin (g/dL)	14.98 ± 0.24	15.20 ± 0.23	0.130	15.19 ± 0.20	15.20 ± 0.17	0.920	0.530	1.000
Hematocrit (%)	44.92 ± 0.68	45.65 ± 0.55	0.130	44.99 ± 0.46	45.17 ± 0.47	0.667	0.933	0.519
Platelet (10^9^/L)	230.5 ± 15.55	232.9 ± 11.83	0.650	220.9 ± 11.01	215.0 ± 9.28	0.413	0.617	0.245
Neutrophils (10^9^/L)	3.32 ± 0.19	3.39 ± 0.24	0.746	2.98 ± 0.21	3.22 ± 0.22	0.333	0.232	0.619
Lymphocytes (10^9^/L)	1.96 ± 0.07	1.96 ± 0.08	0.991	1.90 ± 0.10	1.79 ± 0.10	0.253	0.651	0.234
Monocytes (10^9^/L)	0.37 ± 0.02	0.38 ± 0.02	0.593	0.35 ± 0.02	0.36 ± 0.02	0.689	0.517	0.501
Eosinophils (10^9^/L)	0.22 ± 0.03	0.22 ± 0.03	0.820	0.24 ± 0.03	0.22 ± 0.03	0.236	0.650	0.981
Basophils (10^9^/L)	0.03 ± 0.002	0.04 ± 0.003	0.137	0.03 ± 0.003	0.03 ± 0.002	0.363	0.230	0.159

BMI: body mass index; CK: creatine kinase; GGT: gamma glutamyl-transpeptidase; GOT: aspartate transaminase; GPT: alanine transaminase; HDL: high lipoprotein density cholesterol; LDL: low density lipoprotein cholesterol; VLDL: very low-density lipoprotein. Differences were tested using t-test (with Welch correction when needed) or Kruskal–Wallis tests. Significant differences (*p* < 0.05) and differences that show certain trend (*p* < 0.1) are highlighted in bold. ---, not applicable

**Table 2:** Mean knee temperature (°C) during the visit (IRT0–IRT5) after administration of 2 g of Exten(d) for 30 days to volunteers compared to placebo. Values reported are means ± standard error of the mean.

Variables	Exten(d) (*n* = 13)		*p*	Control (*n* = 15)		*p*	*p*	*p*
	Day 0	Day 30	Intra	Day 0	Day 30	Intra	Inter T0	Inter T1
IRT0	31.05 ± 0.30	29.33 ± 0.46	**0.008**	30.08 ± 0.21	28.63 ± 0.32	**0.001**	**0.013**	0.387
IRT1	30.44 ± 0.27	29.18 ± 0.48	**0.042**	29.86 ± 0.20	28.82 ± 0.30	**0.014**	**0.034**	0.345
IRT2	28.62 ± 0.29	27.69 ± 0.40	**0.043**	27.98 ± 0.27	27.74 ± 0.31	0.434	0.116	0.915
IRT3	31.18 ± 0.26	30.68 ± 0.41	0.247	30.08 ± 0.18	29.80 ± 0.31	0.435	**0.002**	0.098
IRT4	30.15 ± 0.32	29.19 ± 0.45	0.144	29.59 ± 0.30	29.00 ± 0.30	0.101	0.231	0.394
IRT5	27.55 ± 0.33	26.88± 0.52	0.555	27.08 ± 0.31	26.61 ± 0.37	0.468	0.240	0.394

IRT0 = basal round; IRT1 = after acclimation to room temperature; IRT2 = after first biomechanical running test; IRT3 = shortly before incremental test to exhaustion; IRT4 = immediately post-incremental exercise test to exhaustion; IRT5 = after cooling down. Significant differences (*p* < 0.05) are highlighted in bold.

**Table 3:** Changes in biomechanical variables after exercise on a treadmill before and after supplementation.

Biomechanical Variables	Exten(d) (*n* = 13)		*p*	Control (*n* = 15)		*p*	*p*	
	Day 0	Day 30	intra	Day 0	Day 30	Intra	Inter T0	Inter T1
Foot Contact Time (s)	0.248 ± 0.004	0.246 ± 0.003	0.584	0.239 ± 0.004	0.235 ± 0.005	0.247	0.189	**0.098**
Gait Length (m)	2.296 ± 0.035	2.284 ± 0.042	0.555	2.244 ± 0.049	2.261 ± 0.045	0.787	0.504	0.836
Gait Frequency (Hz)	1.345 ± 0.017	1.376 ± 0.017	**0.058**	1.396 ± 0.014	1.402 ± 0.019	0.683	**0.028**	0.328
Hip Flexion at Foot Contact	40.940 ± 1.153	40.251 ± 1.617	0.497	39.276 ± 1.856	38.670 ± 1.403	0.660	0.470	0.465
Max. Hip Extension During Foot Contact (˚)	−0.586 ± 1.123	−1.643 ± 1.444	**0.086**	−0.743 ± 1.373	−1.414 ± 1.204	0.248	0.932	0.903
Max. Hip Adduction During Foot Contact (˚)	9.393 ± 1.241	10.497 ± 1.238	**0.036**	8.903 ± 0.703	9.232 ± 0.933	0.603	0.725	0.415
Max. Hip Rotation During Foot Contact (˚)	−2.992 ± 2.646	−1.560 ± 4.251	0.670	−3.237 ± 2.427	−4.445 ± 1.979	0.474	0.946	0.526
Knee Flexion at Foot Contact	15.708 ± 1.425	15.726 ± 1.802	0.991	18.989 ± 1.680	20.956 ± 2.023	0.194	0.155	**0.068**
Max. Knee Flexion During Foot Contact (˚)	46.272 ± 1.131	45.461 ± 1.061	0.428	47.068 ± 1.331	48.563 ± 1.687	0.263	0.658	0.145
Max. Knee Flexion During Swing Phase (˚)	100.964 ± 2.047	98.662 ± 3.243	0.322	101.995 ± 2.092	101.812 ± 2.261	0.937	0.729	0.423
Max. Knee Valgus During Foot Contact (˚)	−4.331 ± 0.948	−5.453 ± 1.152	0.164	−3.835 ± 0.913	−4.204 ± 1.105	0.484	0.710	0.442
Max. Knee Varus During Foot Contact (˚)	5.383 ± 1.448	5.897 ± 1.741	0.898	4.543 ± 0.804	4.581 ± 0.837	0.917	0.534	0.504
Max. Knee Rotation During Foot Contact (˚)	3.600 ± 1.965	6.639 ± 2.472	0.147	4.593 ± 2.479	5.156 ± 2.501	0.572	0.761	0.679
Foot Flexion at Foot Contact (˚)	8.418 ± 1.022	7.405 ±1.647	0.300	7.488 ± 1.004	8.140 ± 1.352	0.593	0.523	0.730
Max. Foot Extension During Foot Contact (˚)	5.489 ± 0.838	4.759 ± 1.273	0.739	5.840 ± 0.702	6.628 ± 1.277	0.885	0.765	0.504
Foot Supination at Foot Contact (˚)	10.738 ± 1.477	8.473 ± 1.496	0.214	10.749 ± 1.489	10.191 ± 1.795	0.775	0.996	0.477
Max. Foot Pronation During Foot Contact (˚)	−9.089 ± 1.534	−11.431 ± 2.100	0.489	−6.737 ± 1.544	−7.371 ± 2.124	0.950	0.174	0.147
Peak Vertical Reaction Force (N/BW)	2.564 ± 0.049	2.514 ± 0.037	0.397	2.558 ± 0.059	2.541 ± 0.054	0.885	0.800	0.945
Min. Antero-Posterior Reaction Force (N/BW) (Braking)	−0.367 ± 0.011	−0.370 ± 0.017	0.858	−0.360 ± 0.014	−0.364 ± 0.016	0.787	0.596	0.596
Max. Antero-Posterior Reaction Force (N/BW) (Impulse)	0.272± 0.005	0.266 ± 0.006	0.212	0.265 ± 0.009	0.271 ± 0.011	0.464	0.499	0.688

Flexion, adduction, varus, internal rotation and supination have a positive (+) sign, whereas extension, abduction, valgus, external rotation and pronation have a negative (−) sign. N/BW: Newtons/body weight. Differences were tested using t-test (with Welch correction when needed) or Kruskal–Wallis tests. Significant differences (*p* < 0.05) and differences that show certain trend (*p* < 0.1) are highlighted in bold.

## Data Availability

The data presented in this study are available on request from the corresponding author. The data are not publicly available due to ethical reasons.
